# The Potential of Using Brain Images for Authentication

**DOI:** 10.1155/2014/749096

**Published:** 2014-07-10

**Authors:** Fanglin Chen, Zongtan Zhou, Hui Shen, Dewen Hu

**Affiliations:** Department of Automatic Control, College of Mechatronic Engineering and Automation, National University of Defense Technology, Changsha, Hunan 410073, China

## Abstract

Biometric recognition (also known as biometrics) refers to the automated recognition of individuals based on their biological or behavioral traits. Examples of biometric traits include fingerprint, palmprint, iris, and face. The brain is the most important and complex organ in the human body. Can it be used as a biometric trait? In this study, we analyze the uniqueness of the brain and try to use the brain for identity authentication. The proposed brain-based verification system operates in two stages: gray matter extraction and gray matter matching. A modified brain segmentation algorithm is implemented for extracting gray matter from an input brain image. Then, an alignment-based matching algorithm is developed for brain matching. Experimental results on two data sets show that the proposed brain recognition system meets the high accuracy requirement of identity authentication. Though currently the acquisition of the brain is still time consuming and expensive, brain images are highly unique and have the potential possibility for authentication in view of pattern recognition.

## 1. Introduction

Identity authentication is an important task for different applications including access control, ATM card verification, and forensic affairs. Compared with conventional methods (e.g., key, ID card, and password), biometric recognition is more resistant to social engineering attacks (e.g., theft). Biometric recognition is also intrinsically superior that makes it unforgettable. During the past few decades, biometric technologies have shown more and more importance in various applications [1, 2]. Among them, recognition technologies based on fingerprint [3, 4], palmprint [5, 6], iris [7, 8], and face [9, 10] are the most popular.

The brain is the center of the nervous system and the most important and complex organ in the human body. Though different brains may be alike in the way they act and have similar traits, scientists have confirmed that no two brains are or will ever be the same [11]. Both genes (what we inherit) and experience (what we learn) could allow individual brains to develop in distinctly different ways. Recent studies show that the so-called jumping genes, which ensure that identical twins are different, may also influence the brains [12]. All these studies show that the human brain is a work of genius in its design and capabilities, and it is unique. Though brain gray matter will change with age or disease, it shows steadiness in adulthood [13, 14]. The question we are interested in this study is as follows: can we use the brain for identity authentication?

This paper analyzes the uniqueness of human brain and proposes to use the brain for personal identification (authentication). Compared with other biometric techniques, brain recognition is more resistant to forgery (e.g., fake fingerprints [15]) and spoofing (e.g., face disguise [16]). Brain recognition is also more reliable to identify the escapee since one's brain can hardly be modified, whereas other biologic traits may be altered, such as altered fingerprints [17]. Palaniappan and Mandic [18] established a Visual Evoked Potential- (VEP-) based biometrics, and simulations have indicated the significant potential of brain electrical activity as a biometric tool. However, VEP is not robust to the activity of brain. Aloui et al. [19] extracted characteristics of brain images and used them in an application as a biometric tool to identify individuals. Their method just uses a single slice of the brain and thus suffers from the influence of noise. Another drawback of this method is that it only uses intraday scanned brain images, and thus does not consider the interday variation of the acquisition.

The human brain consists of white matter, gray matter, and cerebrospinal fluid. The gyrus is distributed among white matter and gray matter. The shape or the structure of the gray matter of each brain is unique, and it is insensitive to acquisition noise and artifacts [20]. To recognize different person, we can compare the difference in brains' gray matter. Thus, the first step of brain recognition is brain acquisition. There are great developments of various neuroimaging techniques. In this study, we select magnetic resonance imaging (MRI) that is a noninvasive brain imaging technique [21, 22]. After acquiring the brain images, we implement a modified brain segmentation algorithm to extract gray matter from the brain images. Then, a low level feature based matching technique is utilized to investigate individual differences in the whole brain. The approach is applied to two data sets to evaluate the uniqueness of the brain based on recognition (identity authentication). The experimental results illustrate the individual differences of brain images. We found that the individual differences in using the brain for recognition are meticulously distinct and brain matching performs very well in the verification task, indicating that the brain can be used for identity authentication theoretically.

Biometric recognition technologies are generally based on the diversity of biological traits [23]. Our preliminary study that appeared in [24] has confirmed the diversity of the brain. The present paper proposes a practical brain authentication system. (1) It proposes to segment the brain and extract the gray matter shape feature, which is more robust to interclass variation. (2) It introduces an alignment technique for the practical system, and this is the foundation for verification. (3) In the matching stage, we utilize chamfer matching which is robust for matching the shapes of two different images. Comparing results show that shape-based chamfer matching performs better.

The rest of the paper is organized as follows.  Section 2 briefly introduces various acquisition methods of brain images and illustrates the modality used in this study. In Section 3, a modified brain segmentation algorithm is proposed for gray matter extraction.  Section 4 presents the gray matter matching algorithm. Experimental results are presented in Section 5. We finish with summary in Section 6.

## 2. The Brain Acquisition

The first step of brain recognition is brain acquisition. There are great developments in various neuroimaging techniques, such as positron emission tomography (PET), computed tomography (CT), electroencephalograph (EEG), magnetoencephalograph (MEG), and magnetic resonance imaging (MRI). These technologies can generally be classified into two categories: invasive techniques and noninvasive techniques, and invasive techniques cannot be applied to healthy humans. Though CT does not rely on radioactive contrast medium, it does use X-ray, which can be harmful. Both MEG and EEG enjoy high temporal resolution. However, MEG is very expensive and the spatial resolution of EEG is significantly low. By comparison, MRI has both high spatial and high temporal resolution, and it does not require radioactive contrast medium, making it noninvasive.

Due to its high resolution and not requiring radiative contrast medium, MRI has been utilized in brain research since the early 1990s [21]. It has grown rapidly and has become one of the most important brain imaging techniques. The increasing popularity of MRI comes from two characteristics. The first characteristic is that it has no known harmful side effects making it a very patient-friendly and widely accepted technique. Secondly, it produces images with very high anatomical resolution and specificity especially for soft tissues. Therefore, we choose MRI as the modality for this study.

**Figure 1:**
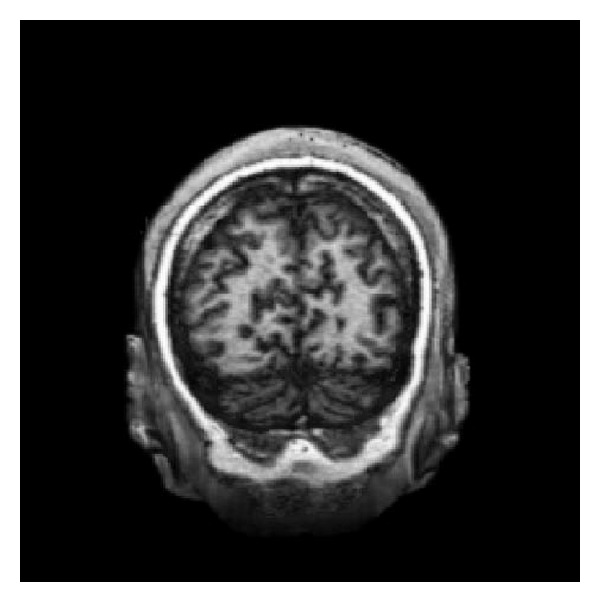
An example of MR image: 3D view from behind with part of hindbrain clipped.

**Figure 2:**
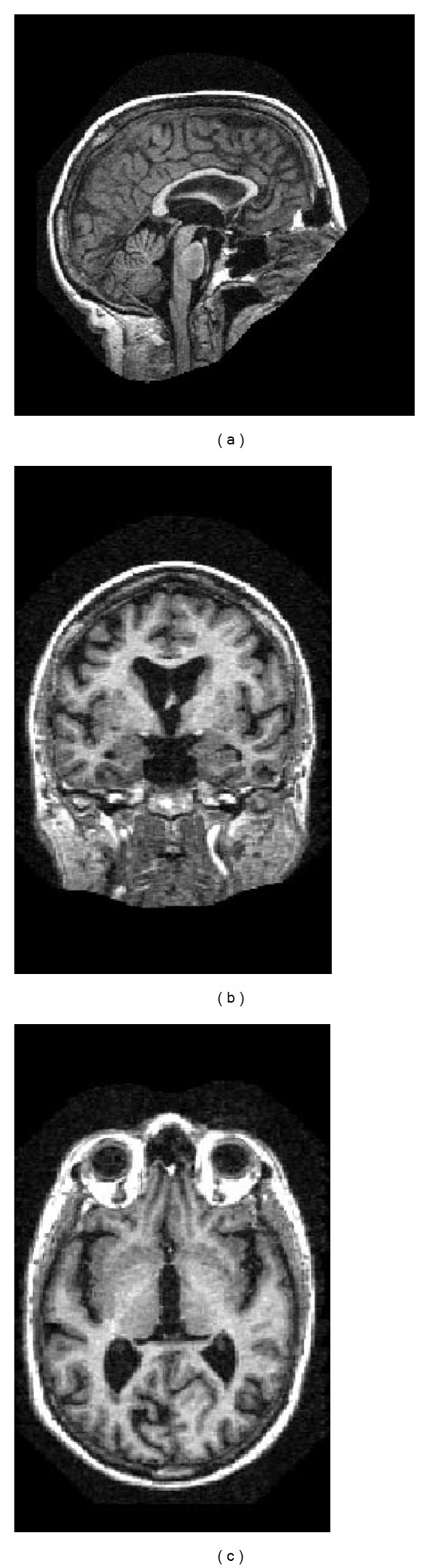
The three projections of the MR image in Figure 1: (a) sagittal, (b) coronal, and (c) axial.

Figure 1 shows an example of MR image (256 × 256 × 128), and its three projections are shown in Figure 2. The sagittal image is viewed as the front (anterior) of the head at the right and the top of the head is shown at the top. This is as if the subject is viewed from the right. The coronal image is viewed as the top (superior) of the head displayed at the top and the left is shown on the left. This is as if the subject is viewed from behind. The axial image is viewed as the front (anterior) of the head at the top and the left is shown on the left. This is as if the subject is viewed from above.

## 3. Feature Extraction

The quality of MR images may be degraded by various factors. Firstly, MRI equipment is likely disturbed by external electromagnetic signals, and they are slightly unsteady. Secondly, in the process of scanning, the testee may have an atom of unavoidably movement. Thirdly, various kinds of physiological signals from human body are correlated to each other, and the SNR (signal-to-noise ratio) of MRI signals is often very low (approximately 2%–5% in 1.5 T MRI signal systems and 5%–20% in 4T MRI signal systems). Under the joint effect of various factors, an MR image is the mixture of several signals. Besides the structure information which we are really interested in, the other information can be treated as noise. Technically speaking, it is impossible to remove the noise completely. Therefore, though there are intergroup analytical procedures based on voxel, they are not suitable for uniqueness analysis. In this recognition problem, due to the hugeness of database, more discriminative features other than voxel are needed. The shape of gray matter is stable for the same person and variant individually [25], and this study utilizes such feature to recognize and analyze the difference in human brain. Thus, we firstly segment the brain image and extract the gray matter then binarize the gray matter to extract the shape feature.

### 3.1. Segmentation

**Figure 3:**
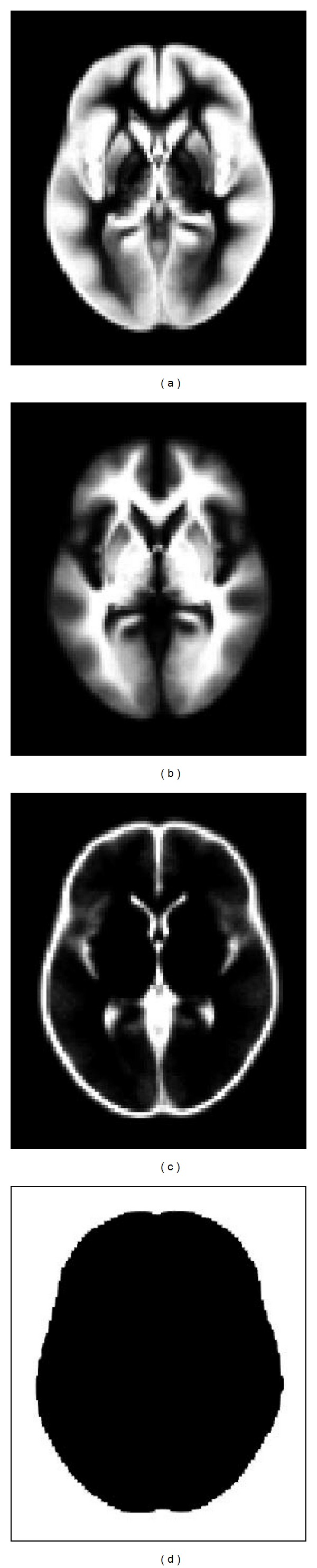
The tissue probability of the template for (a) gray matter, (b) white matter, (c) CSF, and (d) “others,” respectively.

To incorporate both local and global characteristic features of the MR images, we combine the prior information [26] of the human brain and the clustering algorithm for segmentation. The prior information (illustrated in Figure 3) is the approximate knowledge about the spatial distribution of the brain tissues. We choose the maximum likelihood “mixture model” algorithm [27] as the clustering algorithm. The algorithm consists of two steps, which is described as follows.

#### 3.1.1. Determine the Affine Transformation

To determine the affine transformation, we need to find a matrix *M* which can multiply the coordinates of the voxels from the image *I* to the corresponding coordinates of the template *T*. Let [*x*, *y*, *z*] denote the coordinates of *I*, and let [*x*′, *y*′, *z*′] denote the corresponding coordinates of *T*, then the transformation can be illustrated as

(1) $$\begin{array}{c} \left[\begin{array}{c} x^{\prime }\\ y^{\prime }\\ z^{\prime }\\ 1 \end{array}\right]=\left[\begin{array}{cccc} m_{11} & m_{12} & m_{13} & m_{14}\\ m_{21} & m_{22} & m_{23} & m_{24}\\ m_{31} & m_{32} & m_{33} & m_{34}\\ m_{41} & m_{42} & m_{43} & m_{44} \end{array}\right]\times \left[\begin{array}{c} x\\ y\\ z\\ 1 \end{array}\right]. \end{array}$$

Clearly, the fourth row of the transformation matrix *M* is [0 0 0 1]. Assume that the transformation is rigid body, one can obtain a reasonable mapping of most normal brain images to a template image using just a 6-parameter affine transformation, which is formulated as follows:

(2) M=[100p1010p2001p30001] ×[10000cos⁡(p4)sin(p4)00−sin(p4)cos⁡(p4)00001] ×[cos⁡(p5)0sin(p5)00100−sin(p5)0cos⁡(p5)00001] ×[cos⁡(p6)sin(p6)00−sin(p6)cos⁡(p6)0000100001],

where *p*_1_, *p*_2_, and *p*_3_ are the translation parameters to axis *x*, *y*, and *z*, and *p*_4_, *p*_5_, and *p*_6_ are the corresponding rotation parameters. There are no zoom parameters, since the MR images used in this study are scanned by the same modality.

Now, to determine the affine transformation *M*, we need to optimize the parameter set {*p*_1_, *p*_2_,…, *p*_6_}. This can be done by minimizing the sum of square differences between the image *I* and the template *T*. We apply an iterative process to optimize the parameters. Specifically, we use Taylor's theorem to generate a linear approximation to the original optimization problem and solve the approximated problem at each iteration. The chance of finding a local minimum is reduced by smoothing the data (in this study, MR images are convolved with a 5 mm full width at half maximum Gaussian kernel). Thus the method generally converges within a few iterations.

Once the optimization has converged to the final solution, we obtain the rigid body transformation *M* which approximately maps *I* to *T*. The affine transformation matrix *M* is used in the next step, and we can map the prior information *P*_*T*_ to the probability *P*_*I*_ of image *I*.

#### 3.1.2. Segment the Image

Assume that the MR image consists of a number of distinct tissue types, and each type can be seen as a cluster. Every voxel of the image belongs to one of the clusters. We further assume that the voxel intensities of each cluster distribute as a single-argument normal distribution, which can be described by the mean, the variance, and the number of voxels belonging to the tissue type (cluster).

In addition, we have prior knowledge of the spatial distribution of these clusters for the template *T* (see Figure 3 for illustration). These images contain values in the range of 0 to 1, and they represent the prior probability that a voxel being GM, WM, or CSF after the image is transformed to the same space using a 6-parameter affine transformation.

We use four clusters: three for GM, WM, and CSF and one for “others” (including background and scalp, eyes, etc.). Using the 6-parameter affine transformation *M* determined in the previous step, we can map between the space of the image *I* and that of the probability images *P*_*T*_. *P*_*T*_ is a four-dimensional image consisting of *P*_*T*_(1, **x**), *P*_*T*_(2, **x**), *P*_*T*_(3, **x**), and *P*_*T*_(4, **x**) for GM, WM, CSF, and “others,” respectively. **x** is the three-dimensional coordinate. Assume that there are *N* voxels in the image *I*, then the initial probabilities for the *N* voxels can be assigned as follows:

(3) $$\begin{array}{c} p_{nk}=P_{T}\left(k,M^{-1}x_{n}\right), \end{array}$$

where *n* = 1,2,…, *N*, and *k* = 1,2, 3,4 indicate the cluster type.

After the prior probabilities of all voxels for each cluster have been acquired, a modified maximum likelihood “mixture model” algorithm [27] is used to iteratively compute the final probabilities for each voxel. The algorithm is based on the assumption that the intensities of the voxels belonging to each cluster have multivariate normal (Gaussian) distributions. Each distribution can be described by the mean, the variance, and the number of voxels belonging to the corresponding cluster.

The algorithm firstly estimates the distribution parameters, that is, in the normal case, the means *μ*_*k*_, the variance *σ*_*k*_, and the number of voxels belonging to the class *k*, say *m*_*k*_. Secondly, based on the estimated distributions, the probability density functions can be estimated. Finally, it updates and normalizes the probabilities based on the probability density functions. The algorithm repeats iteratively until convergence (or reaching a prespecified iteration number). The algorithm specifying how the belonging probabilities are updated at each iteration is summarized as follows.

(1) *Initialization*: set *t* = 0, *p*_*nk*_(0) = *p*_*nk*_, where *p*_*nk*_ is computed by (3).
(2) Compute the number (*m*) of voxels belonging to each of the 4 clusters as follows:
  (4) $$\begin{array}{c} m_{k}=\overset{N}{\underset{n=1}{\sum }}p_{nk}\left(t\right),\;\left(k=1,2,3,4\right). \end{array}$$
(3) Compute the mean voxel intensities for each cluster (*μ*) as follows:
  (5) $$\begin{array}{c} \mu_{k}=\frac{\sum_{n=1}^{N}p_{nk}\left(t\right)f\left(x_{n}\right)}{m_{k}},\;\left(k=1,2,3,4\right), \end{array}$$
  and *μ*_*k*_ is a weighted mean of the image voxels, where the weights are the current estimated probabilities.
(4) Compute the variance of each cluster in the way similar to the mean:
  (6) $$\begin{array}{c} \sigma_{k}=\frac{\sum_{n=1}^{N}p_{nk}\left(t\right){\left(f\left(x_{n}\right)-\mu_{k}\right)}^{2}}{m_{k}},\;\left(k=1,2,3,4\right). \end{array}$$
(5) Compute the probability density functions for each cluster at each voxel:
  (7) $$\begin{array}{c} g_{nk}=\frac{1}{\sqrt{2\pi \sigma_{k}}}\exp\left(\frac{-{\left(f\left(x_{n}\right)-\mu_{n}\right)}^{2}}{2\sigma_{k}}\right), \end{array}$$
  where *n* = 1,2,…, *N*. and *k* = 1,2, 3,4.
(6) Utilize a new mixture model algorithm:
  (8) $$\begin{array}{c} q_{nk}=g_{nk}p_{nk}\left(t\right), \end{array}$$
  where *n* = 1,2,…, *N*. and *k* = 1,2, 3,4.
(7) Normalize and update the probabilities:
  (9) $$\begin{array}{c} p_{nk}\left(t+1\right)=\frac{q_{nk}}{\sum_{k=1}^{4}q_{nk}}, \end{array}$$
  where *n* = 1,2,…, *N*, and *k* = 1,2, 3,4. Then, the belonging probabilities integrate to unity at each voxel.
(8) Decide to continue or break:
   
**If** (probabilities have converged)
     
**break**.
   
**Else**
     
*t* = *t* + 1,** Goto** Step (2).
   
**Endif**

The algorithm is repeated iteratively and the parameters (*μ*_*k*_, *σ*_*k*_, and *m*_*k*_, *k* = 1,2, 3,4) fit in the distribution better and better. Meanwhile, the belonging probabilities (*p*_*nk*_, *n* = 1,2,…, *N*, *k* = 1,2, 3,4) change steadily to reflect the real segmentation. Though the values of *m*_*k*_, (*k* = 1,2, 3,4) are computed from the probabilities, they do not describe the distributions and the probabilities themselves. The probabilities are iteratively computed from the a priori probability images, and they are more accurate to reflect the distributions. Thus the improvement of the “mixture model” tends to make the algorithm have better convergence. The values of *p*_*nk*_ (*n* = 1,2,…, *N*, *k* = 1,2, 3,4) are in range [0,1], and most of them may finally converge to one of the two endpoints: 0 or 1.

**Figure 4:**
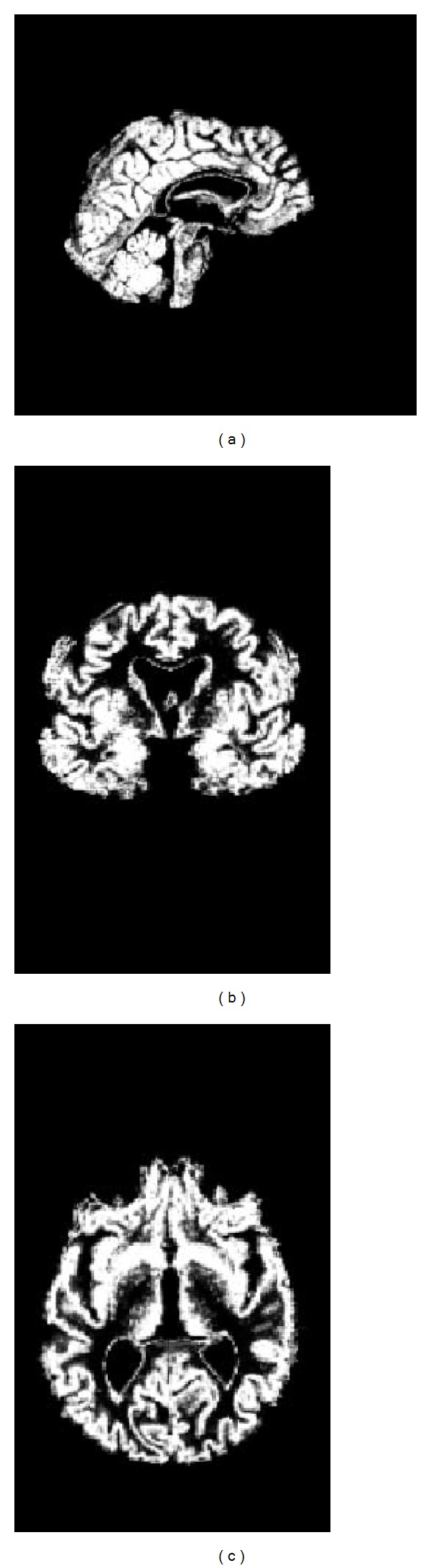
Segmentation results (gray matter) by the proposed method: (a) sagittal; (b) coronal; and (c) axial.

Figure 4 shows the segmentation results (gray matter) of one brain image estimated by the proposed method. The results show that the algorithm can segment the MR images with satisfactory precision for matching.

### 3.2. Extracting Gray Matter

Even for the same subject, if the MR images are scanned at different time, the gray matter intensities of these images will be different to each other. This is due to the influence of various factors, including endogenic (e.g., blood pressure and brain activity) and exogenic ones (e.g., the electronics of the MR system and the external environment). Thus, we cannot match two MR images directly using the gray matter intensities. Though the intensities may change at different scanning times, the structure of the tissues is stable and insensitive to acquisition noise and artifacts. Inspired by this property, we propose to extract the gray matter structure first and then conduct the matching.

Let *P*_*g*_ denote the probabilities for the gray matter cluster. The greater value of *P*_*g*_(**x**) indicates the higher probability that voxel **x** belongs to the gray matter cluster. We extract the gray matter structure via segmenting *P*_*g*_. After the segmentation of *P*_*g*_, we get a binarized image that represents the structure of the gray matter using Otsu's method [28]. Since the images scanned on different visits may be scaled quite differently, the binarized image is more robust than *P*_*g*_.

## 4. Matching Gray Matter

To compare two binarized gray matter images *B*_1_ and *B*_2_ (extracted from the two MR images, denoted as *I*_1_ and *I*_2_, resp.), the first step is to align the corresponding two images. In this study, an alignment-based matching algorithm is implemented. Matching by alignment has received a great deal of attention during the past decades [29, 30], since it is simple in theory, efficient in discrimination, and fast in speed. The proposed alignment-based matching algorithm decomposes the gray matter matching into two stages.

1. Alignment stage: where transformations such as translation and rotation between *B*_1_ and *B*_2_ are estimated and one of the two gray images is aligned with the other one according to the estimated parameters;
2. matching stage: where the similarities between *B*_1_ and *B*_2_ are evaluated by chamfer matching and an linear transformation is used to normalize the similarity scores.

### 4.1. Alignment of Gray Matter Images

In order to align the two gray matter images, the parameters of translation and rotation between the two images are needed to be estimated. In other words, the affine transformation matrix *M*_*b*_ mapping *B*_1_ to *B*_2_ is needed. Let *B*_1_′ denote the aligned gray matter image from *B*_1_ by *M*_*b*_. The transformation matrix should minimize the error between *B*_1_′ and *B*_2_. Ideally, *B*_1_ and *B*_2_ can be treated as two sets of stereoscopic points and can be aligned completely by two corresponding point pairs. A true alignment between two point patterns can be obtained by testing all possible corresponding point pairs and selecting the optimal one. However, due to the presence of noise and deformations, the points of *B*_1_ cannot always be aligned exactly with respect to those of *B*_2_. Moreover, the number of points is always very large (e.g., for a 256 × 256 × 128 image, the number is 8388608 ≈ 10^7^), which leads to a prohibitively large number of possible correspondences. Therefore, an alignment by corresponding point pairs is not practical even though it is feasible.

In the segmentation stage, we have determined the transformation matrix. We can make use of these results to finish the alignment. Let *M*_1_ and *M*_2_ denote the transformation matrix from *I*_1_ and *I*_2_ to the template *T*, respectively. Then the transformation mapping from *I*_1_ to *I*_2_ is

(10) $$\begin{array}{c} M_{12}=M_{1}M_{2}^{-1}. \end{array}$$

Thus we can align the two binarized gray matter images *B*_1_ and *B*_2_ without estimating the transformation matrix. Though this strategy uses a transitional image *T* as the bridging, it preserves the alignment accuracy since it exploits the original plenty voxel information of the images.

### 4.2. Matching

Matching methods can be mainly divided into three classes [31]: (1) algorithms that use the image pixel (voxel) values directly; (2) algorithms that use low-level features such as edges and corners; and (3) algorithms that use high-level features. Methods which use the image pixel values directly, such as correlation methods, are sensitive to shift and rotation between images, thus they are not widely used. The drawback of high-level matching methods is that high-level features need to be extracted first and identified, which is a rather difficult task. We treat gray matter based brain matching as a problem of low-level matching. Compared with the other two methods, low-level matching method is more robust than methods that use the image pixel values directly, and its features are easier to extract than high-level matching methods.

Denote *B*_1_′ and *B*_2_′ as the aligned binarized gray matter images from *B*_1_ and *B*_2_, respectively. Actually, *B*_2_′ is the same as *B*_2_, since it only needs to transform *B*_1_ to *B*_1_′. Here we use *B*_2_′ only for looking good in deduction. Among all the low-level matching methods, chamfer matching is a state-of-art algorithm. Chamfer matching is widely used to match shapes in two different images [32, 33]. In the chamfer matching step, the difference between two aligned gray matter images, *B*_1_′ and *B*_2_′, is computed as shown below:

(11) $$\begin{array}{c} d=\underset{x}{\sum }\underset{y}{\min}\left\{\text{dis}\left(x,y\right),\;\;\text{with}\;\;B_{1}^{\prime }\left(x\right)=1,\;\;B_{2}^{\prime }\left(y\right)=1\right\}, \end{array}$$

where **x** and. **y** can be any possible three-dimensional coordinates with *B*_1_′(**x**) = 1 and. *B*_2_′(**y**) = 1, respectively. dis(**x**, **y**) is the Euclidean distance between **x** and **y**. A smaller *d* means a higher probability that the two MR images come from the same subject.

After *d* is computed, the similarity (final score *s*) between the two gray matter images, *B*_1_ and *B*_2_, is normalized from *d* by

(12) s=−100−0dmax⁡−dmin⁡×(d−dmin⁡)+100=(1−d−dmin⁡dmax⁡−dmin⁡)×100,

in which *d*_max⁡_ and *d*_min⁡_ (obtained by training) represent the possible maximum and minimum value of all differences *d*, respectively. Equation (12) normalizes the scores to interval [0,100], where 100 means full matching while 0 stands for mismatching.

## 5. Experiments

The experiments are conducted on two data sets:* OAS1* and* OAS2*. The data sets can be downloaded from the Open Access Series of Imaging Studies (OASIS) website [34]. There are 416 (half of which are selected for training set) persons in* OAS1* and 150 persons in* OAS2*. For each person, *T*_1_-weighted structural magnetization-prepared rapid gradient echo (MP-RAGE) images are obtained with the following parameters: TR = 9.7 ms, TE = 4.0 ms, slice thickness = 1.25 mm, slice  number = 128, flip  angle = 10°, and in-plane resolution = 256 × 256 (1 mm × 1 mm). There are many persons that participate in at least two separate visits (necessary for testing system's robustness to interday variation) on which MRI data are obtained.

### 5.1. Matching

Each brain in the database is matched with the other brains. Genuine matching indicates that two matching brain images are acquired from the same subject, while imposter matching indicates that two matching brain images are scanned from different individuals. The genuine matching pairs are generated by images from the same subject but different visits.

**Figure 5:**
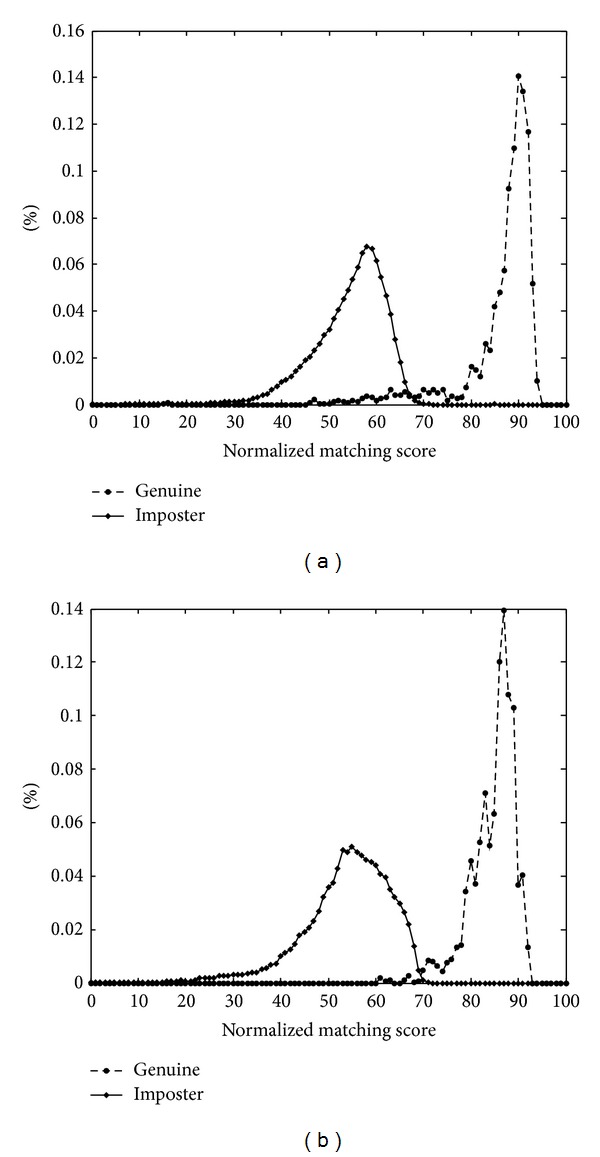
Distributions of genuine and imposter matching scores; vertical axis represents distribution of matching scores in percentage. (a) Distribution of matching scores on* OAS1*. (b) Distribution of matching scores on* OAS2*.

The distributions of normalized genuine and imposter matching scores are shown in Figure 5. It can be observed from this figure that two peaks exist in the distribution of matching scores. One peak is located at a value near 58, corresponding to the imposter matching scores. The other pronounced peak resides at the value 88 and is associated with the genuine matching scores. This result indicates that our algorithm is capable of differentiating brains at a high rate of accuracy by selecting an appropriate value of the threshold.  Table 1 shows the true acceptance rates and false rejection rates with different threshold values. The false rejection rate is defined as the percentage of genuine pairs with their matching scores below the threshold value. The result illustrates that the proposed verification system can gain very high true acceptance rates at low false rejection rates, indicating the possible use of brain images for authentication. Compared with Aloui's method [19], our algorithm can reach to a maximum accuracy [19] of 99.46%, which is much higher than the maximum accuracy of 98.25% in Aloui's method. The main shortage of Aloui's method is that it just uses one slice of the brain image, and it is hard to extract the same slice at different scans.

### 5.2. Comparing with Pixel-Based Matching

**Figure 6:**
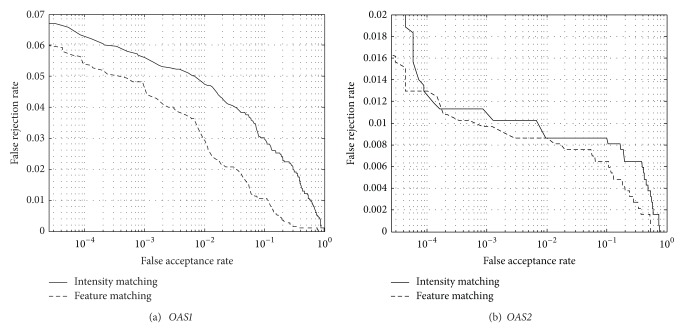
The ROCs of pixel-based (intensity) matching and the proposed feature-based matching.

The proposed matching algorithm is based on feature matching. To compare it with the pixel-based (intensity) matching, we also conduct the matching experiment on the same database. The pixel-based matching used the intensity directly for evaluating the similarity between brain images.  Figure 6 shows the receiver operating curves (ROCs) plotting false acceptance rate (FAR) versus false rejection rate (FRR) of pixel-based matching scheme (solid line) and the proposed scheme (dash line). FRR is defined as the percentage of imposter matches in all genuine pairs, while FAR is defined as the percentage of genuine matches in all imposter pairs. The results show that the gray matter matching can largely improve the performance, compared with the intensity matching. FRR can be reduced a lot by matching the gray matter against the intensity-based matching. The equal error rates (EERs) of the gray matter matching on the two data sets are 2.13% and 0.86%, which are much lower than those of the intensity matching (3.88% and 0.92%), respectively. This also validates the effectiveness of the proposed algorithm.

## 6. Conclusion and Future Work

We have analyzed the uniqueness of the brain and designed a verification system for identity authentication. The system operates in two stages: gray matter extraction and gray matter matching. A modified brain segmentation algorithm was developed. A binarization scheme was conducted to extract the gray matter which can improve the performance of matching. An alignment-based matching algorithm was proposed for gray matter matching. Experimental results show that our system achieves excellent performance in the testing database. Though currently the acquisition of the brain is still time consuming and expensive for users, we believe that the brain will be one of the members of biometric technologies in the future when the acquisition technique is developed.

Aloui's method only uses a slice and can reach a fair performance. This enlightens us that we may decrease the resolution of MRI image and then can speed up the acquisition and processing step. Thus our future work will focus on two aspects. (1) Decrease the resolution of MRI image by sampling, and then analyse the performance. (2) Use low resolution device which can be of low cost and fast to obtain the MRI image and make the system applicable.

## Figures and Tables

**Table 1a:** (a) *OAS1*

Threshold value	True acceptance rate	False rejection rate
68	99.814%	5.31%
69	99.938%	5.76%
70	99.984%	6.17%
71	99.999%	6.70%
72	99.999%	7.32%

**Table 1b:** (b) *OAS2*

Threshold value	True acceptance rate	False rejection rate
68	98.771%	0.73%
69	99.671%	0.77%
70	99.926%	1.02%
71	99.994%	1.57%
72	100.000%	2.82%

## References

[1] Chen F, Huang X, Zhou J (2013). Hierarchical minutiae matching for fingerprint and palmprint identification. *IEEE Transactions on Image Processing*.

[2] Al-juboori AM, Wu X (2014). Palm vein veri fication using multiple features and locality preserving projections. *The Scientific World Journal*.

[3] Feng J, Ouyang Z, Cai A (2006). Fingerprint matching using ridges. *Pattern Recognition*.

[4] Chen F, Feng J, Jain AK, Zhou J, Zhang J (2011). Separating overlapped fingerprints. *IEEE Transactions on Information Forensics and Security*.

[5] Zhang D, Guo Z, Lu G, Zhang L, Zuo W (2010). An online system of multispectral palmprint verification. *IEEE Transactions on Instrumentation and Measurement*.

[6] Liu H, Sun D, Xiong K, Qiu Z (2014). Palmprint based multidimensional fuzzy vault scheme. *The Scientific World Journal*.

[7] Hollingsworth KP, Bowyer KW, Flynn PJ (2011). Improved iris recognition through fusion of hamming distance and fragile bit distance. *IEEE Transactions on Pattern Analysis and Machine Intelligence*.

[8] Chou C-T, Shih S-W, Chen W-S, Cheng VW, Chen D-Y (2010). Non-orthogonal view iris recognition system. *IEEE Transactions on Circuits and Systems for Video Technology*.

[9] Dantcheva A, Chen C, Ross A Can facial cosmetics affect the matching accuracy of face recognition systems?.

[10] Cament LA, Castillo LE, Perez JP, Galdames FJ, Perez CA (2014). Fusion of local normalization and gabor entropy weighted features for face identification. *Pattern Recognition*.

[11] Gage FH, Muotri AR (2012). What makes each brain unique. *Scientific American*.

[12] Choi C (2005). Jumping genes in the brain. *Genome Biology*.

[13] Loftus EF, Loftus GR (1980). On the permanence of stored information in the human brain. *American Psychologist*.

[14] Fraley RC (2002). Attachment stability from infancy to adulthood: meta- analysis and dynamic modeling of developmental mechanisms. *Personality and Social Psychology Review*.

[15] Baldisserra D, Franco A, Maio D, Maltoni D (2005). Fake fingerprint detection by odor analysis. *Advances in Biometrics*.

[16] MSNBC Man boards flight in “elderly” disguise. http://www.nbcnews.com/id/40026355/ns/.

[17] Yoon S, Feng J, Jain AK (2012). Altered fingerprints: analysis and detection. *IEEE Transactions on Pattern Analysis and Machine Intelligence*.

[18] Palaniappan R, Mandic DP (2007). Biometrics from brain electrical activity: a machine learning approach. *IEEE Transactions on Pattern Analysis and Machine Intelligence*.

[19] Aloui K, Nait-Ali A, Naceur MS A novel approach based brain biometrics: some preliminary results for individual identification.

[20] Westbrook C, Roth C (2011). *MRI in Practice*.

[21] Ogawa S, Menon RS, Tank DW (1993). Functional brain mapping by blood oxygenation level-dependent contrast magnetic resonance imaging. A comparison of signal characteristics with a biophysical model. *Biophysical Journal*.

[22] Wang JJ, Bensmail H, Gao X (2013). Joint learning and weighting of visual vocabulary for bag-of-feature based tissue classification. *Pattern Recognition*.

[23] Jain AK, Bolle R, Pankanti S (2005). *Biometrics: Personal Identification in Networked Society*.

[24] Chen F, Su L, Liu Y, Hu D (2013). Confirming the diversity of the brain after normalization: an approach based on identity authentication. *PLoS ONE*.

[25] Gilaie-Dotan S, Harel A, Bentin S, Kanai R, Rees G (2012). Neuroanatomical correlates of visual car expertise. *NeuroImage*.

[26] Ashburner J, Friston KJ (2005). Unified segmentation. *NeuroImage*.

[27] Bishop CM (2006). *Pattern Recognition and Machine Learning*.

[28] Otsu N (1979). A threshold selection method f rom gray-level histograms. *IEEE Transactions on Systems, Man, and Cybernetics*.

[29] Li W, Zhang L, Zhang D, Lu G, Yan J Efficient joint 2D and 3D palmprint matching with alignment refinement.

[30] Myronenko A, Song X (2010). Point set registration: coherent point drifts. *IEEE Transactions on Pattern Analysis and Machine Intelligence*.

[31] Zhou J, Chen F, Wu N, Wu C (2009). Crease detection from fingerprint images and its applications in elderly people. *Pattern Recognition*.

[32] Liu M-Y, Tuzel O, Veeraraghavan A, Chellappa R Fast directional chamfer matching.

[33] Demirci MF Efficient shape retrieval under partial matching.

[34] Buckner R, Neuroinformatics Research Group (NRG), Biomedical Informatics Research Network (BIRN) (2012). * Open Access Series of Imaging Studies (Oasis)*.

